# Study of the variability of scapular inclination and the glenoid version - considerations for preoperative planning: clinical-radiological study

**DOI:** 10.1186/s12891-016-1381-4

**Published:** 2017-01-14

**Authors:** Petr Fulin, Martin Kysilko, David Pokorny, Radek Padr, Nikola Kasprikova, Ivan Landor, Antonin Sosna

**Affiliations:** 1 Orthopaedic Clinic 1st Faculty of Medicine Charles University and Motol University Hospital, V Uvalu 84, Prague, postal code 15006 Czech Republic; 2 Clinic of Imaging Methods 2nd Faculty of Medicine Charles University and Motol University Hospital, Prague, Czech Republic; 3 Institute of Biophysics and Informatics, First Faculty of Medicine, Charles University in Prague, Prague, Czech Republic

**Keywords:** Glenoid, Version, Inclination, Scapula, Preoperative planning, Arthroplasty, Angle

## Abstract

**Background:**

Preoperative planning with the aid of imaging methods is a principal factor in successful surgery on the shoulder. This work aims to evaluate the variability of glenoid version, spiralling twist and scapular inclination in relation to the frontal axis. Studies focusing on measuring the variability of scapular inclination in the standardised rest position are lacking in the literature.

**Methods:**

We evaluated 104 CT scans of the shoulder. We measured the glenoid version with respect to the scapular axis at three levels. We measured the scapular inclination angle in relation to the sagittal plane and we determined scapular inclination in relation to the frontal axis. Statistical evaluation was performed using the marginal linear model and parameters were estimated using the generalised least squares method, which enables the dependency of measurements performed on the same subject to be taken into consideration.

**Results:**

The highest values of retroversion are attained by the glenoid in the cranial section (average -9.96°, range -29.7 to +13.2°). Proof of the spiralling twist is the decline in retroversion at the centre of the glenoid (average -2.09°, range -16.7 to +11.6°).

Retroversion decreases further in the inferior direction (average -0.5°, range -20.9 to +17.5°). The average thoracoscapular angle is 45.46°, ranging from 13.1 to 69.0°. The average scapular inclination in relation to the frontal plane is 44.54°, ranging from 21.0 to76.9°.

**Conclusions:**

During preoperative planning, the surgeon should take into consideration not only the glenoid version in relation to the scapular axis, but also the value of the scapular inclination so as to eliminate possible surgical errors, optimise prosthesis implantation and thus decrease the risk of functional restrictions of the joint.

**Clinical trial registration:**

Ethics Committee for Multi-Centric Clinical Trials (EK-554/14,29thApril 2014).

## Background

Preoperative planning using imaging methods is an indispensable step in the case of surgery involving the glenoid region, especially arthroplasty and the correction of joint instability. Most studies involving both normal and degeneratively altered shoulder joints focus on measuring the glenoid version in relation to the scapular axis. The aim is to achieve correct positioning of the glenoid or prosthesis component so that this best corresponds to both anatomical and actual biomechanical conditions. Studies measure glenoid retroversion in relation to the scapular axis based on cadaveric sections [1], and on radio-imaging documentation (X-ray, CT, MRi) [2–5]. CT scans are most frequently used to determine the position of the glenoid. According to available literature, X-ray based measurement is not as accurate. Other studies focus on measuring the degree of glenoid erosion using CT or MRI methods [6]. The importance of CT measurement for preoperative planning of correct retroversion was also confirmed by Ganapathi [7]. Some studies also measure the relationship between the glenoid and proximal humerus [8], with the aim of improving the centering of the prosthetic implant. In concurrence with our observations, a number of studies note that in the superior-to-inferior direction, there is a decrease in native glenoid retroversion within the frame of its spiral torsion [9]. Glenoid version is thus a key factor in surgical procedures involving the glenoid, especially in the case of total anatomical replacement or reverse shoulder replacement.

It has not been possible, in surgery, to determine the exact position of the glenoid or rather its true orientation in space. Peri-operative orientation may be distorted by a number of factors, such as the patient’s position on the operating table, the positioning of the patient’s head, obesity etc.

**Fig. 1:**
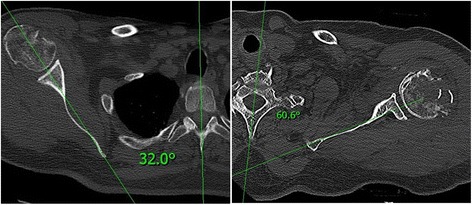
Variability of scapular inclination (thoracoscapular angle - TSa) in relation to the sagittal plane in two different patients

Figure 1 demonstrates the difference in scapular inclination with respect to relatively identical glenoid versions. The variability of scapular inclination in relation to the thorax may be an important factor that distorts the final peri-operative evaluation of glenoid retroversion and may thus affect the surgical outcome. Various works deal with changes in scapular inclination in relation to the thorax during the locomotor cycle [10–12].

There is a lack of studies in the literature measuring the variability of this inclination in a standardised rest position. Meaning it position on the CT scan in supination is the closest position of the patient on the operating table. Patient lies on back with upper limb loosely at your sides with supination of the forearm. Correct peri-operative evaluation of the glenoid version may have a fundamental effect on the surgical outcome - especially in the case of total shoulder joint replacement surgery. Hypothesis of the study is that the actual position of the glenoid in relation to the patient’s frontal plane is affected by two issues - the true version of the native glenoid in relation to the scapular axis and the position of the scapula in relation to the trunk (the thoracoscapular angle – TSa). The aim of this work was to perform an anatomical-radiological study using CT scans to determine the variability of native glenoid retroversion, its spiralling twist and the variability of scapular inclination in relation to the frontal and sagittal planes.

## Methods

This study was approved (issues favourable opinion) at 29^th^ April 2014 by Ethics Committee for Multi-Centric Clinical Trials (Reference No.: EK-554/14). Written informed consent was obtained from all of study subjects.

### Study population

We evaluated a total of 104 CT scans of the shoulder from 89 patients in the period from August 2010 until November 2014. In 14 patients, a CT of the contralateral healthy shoulder was also performed as part of a single exam (CT of the chest and both shoulders). In 17 patients, the CT was performed bilaterally. The average age of the patients at the time of the scan was 59.53 years, ranging from 19 to 97 years with a standard deviation (SD) of 17.37. Women were represented in 36 cases and men in 53 cases. Both the right and left shoulders were evaluated in 52 cases.

### Inclusion and exclusion criteria

The study included patients who do not primarily damaged glenoid (by trauma or abrasion) and who have no deformity of the chest or spine. The study did not include patients suffering from muscular or neurological disorder. The most frequent indications of the CT scan included fractures of the proximal humerus (46 patients), omarthritis (23 patients), pseudoarthrosis of the proximal humerus (six patients), instability (five patients), prior stabilisation (two patients), prior osteosynthesis of the clavicle (two patients), and cystic changes of the glenoid (two patients). We excluded up front CT exams where it was impossible to clearly determine the medial border of the scapula due to significant rotation in the coronal plane, due to the possible distortion of the result of glenoid retroversion and scapular inclination measurements [2].

### CT examination

**Fig. 2:**
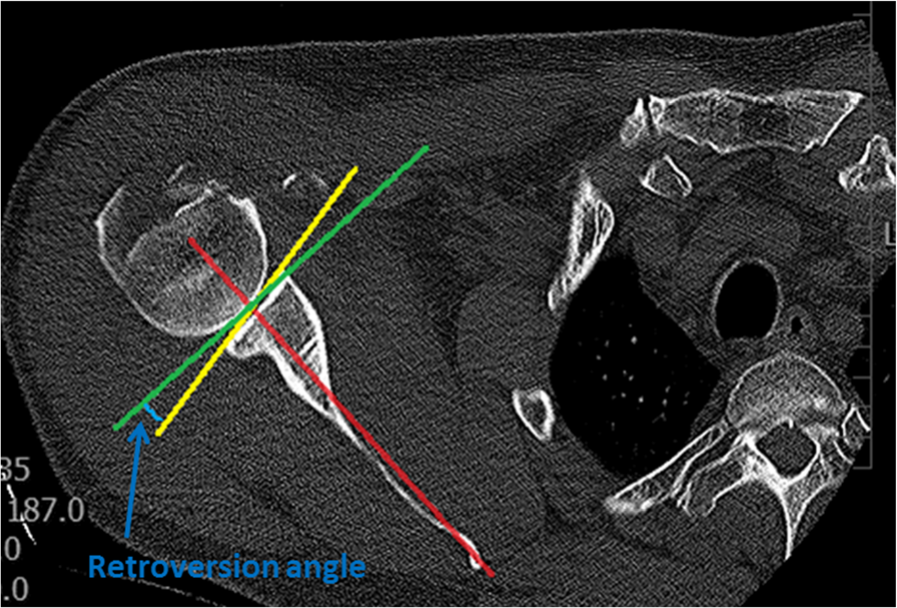
Diagram of glenoid retroversion angle measurement. The scapular axis (Friedman line) is marked in *red*. The line perpendicular to the scapular axis passing through the centre of the glenoid is marked in *green* and the line connecting the rims of the glenoid is marked in *yellow*. The angle of glenoid version is marked in *blue*

The examination took place in the supine position with the head slightly supported so that it maintained a neutral position, with the limbs lying freely alongside the body in a neutral position. The CT was performed using the Siemens Somatom Definition 7740769 (Siemens AG Medical Solutions, DE) and Toshiba Aquillion TSX 101-A (Toshiba Medical Systems Europe NL) devices, with reconstruction and slice thickness of 1.5 mm. We used the xVision – xViewer v.2.7.1 program (Vidis s.r.o., Prague, CZ) for our measurements. We used the Friedman method to measure retroversion [9, 13, 14]. The axis of the scapula passed through the centre of the glenoid and the medial border of the scapula vertex (Fig. 2). The line perpendicular to this passing through the centre of the glenoid then represented the reference plane in relation to which glenoid torsion was measured (Fig. 2). The dorsal inclination of the glenoid was designated as retroversion and the angle was expressed as a negative number. The ventral inclination was designated as anteversion and the angle was expressed as a positive number. We measured glenoid version on three levels. The first measurement was performed in the area of the cranio-caudal centre of the glenoid (half the distance to the proximal and caudal border of the glenoid), and then measurements were taken 9.5 mm (6 CT slices) above and below this level (designation as version middle – VM, version below – VB and version above – VA). This is based on our experience with observation of the size and shape of the glenoid in vivo on dry anatomical preparations. Within the framework of glenoid version measurement, we had to exclude six measurements from the sample due to dorsal abrasion of the glenoid associated with omarthritis (type B2) these would have distorted the overall results.

### Data analysis

**Fig. 3:**
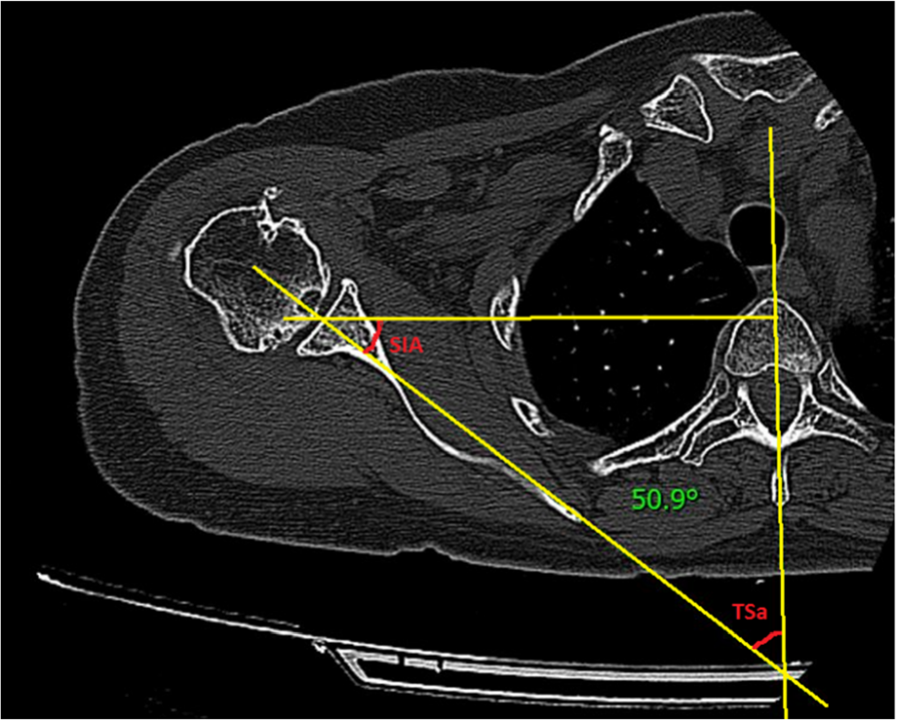
Graphical depiction of the angles determining scapular inclination (SIA – scapular inclination angle, TSa –Thoracoscapular angle)

The principal aim of these measurements was to determine the angle between the scapular axis and the reference plane passing through the centre of the vertebral body, the spinal canal and the vertex of the spinous process of the corresponding vertebra (thoracoscapular angle – TSa) (Fig. 3). This angle was measured at the level of the cranio-caudal centre of the glenoid. For practical purposes, we calculated again the TSa with respect to the scapular inclination angle – the SIa, which is the angle formed by the axis of the scapula and the frontal plane (Fig. 3). Results of measurements two main investigators were differed minimally. Statistical evaluation was performed using the marginal linear model and parameters were estimated using the generalised least squares method, which enables the dependency of measurements performed on the same subject to be taken into consideration. The 5% level of significance or the 95% confidence interval was chosen to assess statistical significance.

## Results and discussion

The goal of this study was to perform an anatomical-radiological study using CT scans to determine the variability of native glenoid retroversion, its spiralling twist and the variability of scapular inclination in relation to the frontal and sagittal planes.

**Fig. 4:**
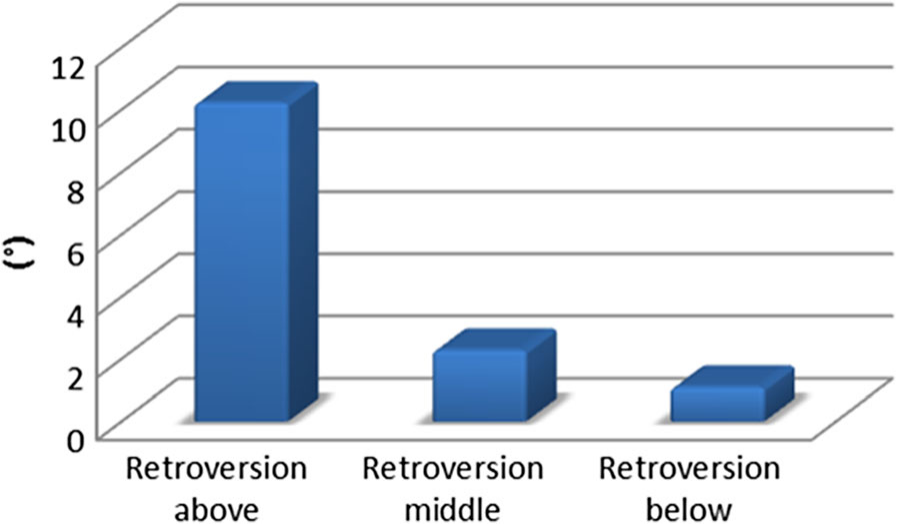
Glenoid retroversion at various levels of measurement. The *y* axis depicts the degree of retroversion in °. The numerical values of retroversion on the *y* axis correspond to the negative numbers from the measurement results

The average results of glenoid version measurement show that most of the glenoids were in retroversion. The glenoid attains the highest values of retroversion in its cranial section (average -9.96°, range -29.7 to +13.2°, SD = 8.94). The decrease in retroversion at the centre of the glenoid is proof of the spiralling twist (average -2.09°, range -16.7 to +11.6°, SD = 6.67). Retroversion further decreases caudally (average -0.5°, range -20.9 to +17.5°, SD = 6.95). The mean value (corresponding to the weighted average) of VA (version above) is -9.55, with a 95% confidence interval of -11.06 to -8.05. The mean value of the difference between the VM (version middle) value and the VA (version above) value is 7.87, with a 95% confidence interval of 6.71 to 9.03. The mean value of the difference between the VB (version below) value and the VA (version above) value is 9.46, with a 95% confidence interval of 8.3 to 10.62. The difference between the VA (version above) value and the VM (version middle) value is statistically significant (*p*-value < 0.0001). The difference between the VM (version middle) value and the VB (version below) value is statistically significant (*p*-values = 0.0075) and the difference between the VA (version above) and VB (version below) values is also statistically significant (*p*-value < 0.0001). These statistical outputs demonstrate the decreasing degree of retroversion in the superior-to-inferior direction (from -9,96° in the proximal part (VA) to -2,09° in the middle (VM) to -0,5° in caudal part (VB). The difference between the value of version above (VA) and version middle (VM) is statistically significant (*p*-value <0.0001) as well as the difference between the value of version - middle (VM) and version - below (VB) is statistically significant (*p*-value = 0.0075). Figure 4 (Fig. 4) depicts the measurement of retroversion.

**Fig. 5:**
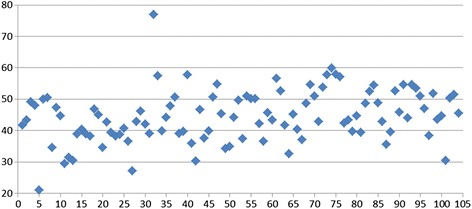
Scapular inclination angle (SIa). Individual measurements on the *x*-axis and degrees on the *y*-axis

The average thoracoscapular angle TSa is 45.46°, range 13.1–69.0° with a standard deviation of 8.29. The average scapular inclination angle SIa is 44.54°, range 21.0–76.9° and a standard deviation of 8.29°. Figure 5 (Fig. 5) depicts the individual measurements of the SIa angle. According to the statistical evaluation, TSU mean value is 45.7, with 95% confidence interval of 43.9 to 47.4. The mean value is significantly different from zero (*p*-value <0.0001). SIA mean value is 44.3, with 95% confidence interval of 42.6 to 46.1. The mean value is significantly different from zero (*p*-value <0.0001).

The results of measurements within each age group are shown in Table 1. The data indicate a slight reduction for SIa with increasing age. Exception is the category under 30 years. Glenoid version is based on available data (Table 1) depends on the patient’s age.

**Table 1:** Results of measurement (SIa (scapular inclination angle), TSa (thoracoscapular angle), glenoid version (VA – version above, VB – version below, VM – version middle) within different age groups.)

Age	TSa	SIa	VA	VM	VB
≤30	45,2	44,8	−9,0	−0,8	1,4
31–40	41,6	48,4	−10,1	−2,3	−2,0
41–50	41,1	48,9	−10,7	−5,8	−7,0
51–60	44,2	45,8	−9,2	−1,7	−1,0
61–70	46,1	43,9	−10,9	−1,4	0,5
71–80	48,3	41,7	−10,9	−2,3	0,0
≥ 81	49,4	40,6	−2,2	2,1	9,4

The average values of retroversion differ in individual studies. Bouchaib [9] cites glenoid retroversion in the widest anterior-posterior dimension as being 4.04°. In the region of the notch of the anterior glenoid rim, this study reports an average retroversion of 6.85° and in the region of the coracoid process, retroversion of up to 11.9°. The results equally demonstrate the spiral configuration of the glenoid. It must be noted however that retroversion is not measured at the same sites of the glenoid. Matsumura [15] performed measurements on 410 CT scans with an average glenoid retroversion in the middle section of 1° ± 3°, which correlated with our results of an average retroversion of 2.09°. Roleau [14] reported an average retroversion of 10.43° according to the Friedman method, however, he does not specify whether this relates to the geometric centre of the glenoid or to the site of the widest glenoid diameter. Nonetheless, we must take into account the measurements in the group of patients with omarthrosis, where abrasion of the dorsal rim of the glenoid is apparent. Hoenecke [15] points out the necessity of measuring glenoid retroversion and the degree of dorsal rim abrasion with the aid of 3D computer reconstruction. This is also confirmed by the study conducted by Beuckelaers [16] who used CT scans to measure the degree of glenoid erosion in type B glenoid abrasion. Moineau [17] also focused on the shape of the glenoid in patients with osteoarthritis, using 3D CT scans. Conversely, Budge [18] demonstrated that measurement of glenoid retroversion using 2D and 3D CT scans did not suggest any differences.

In all patients enrolled in our study, the CT exam was indicated for reasons other than the purposes of this study. None of the patients were deliberately subjected this study was approved by the ethics committee extra X-ray doses.

The Bouchaib [9] study also confirms our findings that retroversion decreases in the superior-to-inferior direction – proof of glenoid spiralling twist [19, 20], which to date is not taken much into consideration when planning total joint replacements.

A number of studies deal with the change in the position of the scapula induced by movement or by the mutual position of the glenoid and proximal humerus during the locomotor cycle [10–12]. Studies measuring the variability of this inclination in the standardised rest position are lacking in the literature. In fact, scapular inclination may have a fundamental effect on the peri-operative assessment of glenoid retroversion as depicted, for example in Fig. 1. For example, a difference of 30° in scapular inclination leads to a visually inexact assessment of glenoid retroversion during surgery, especially in view of the visual inaccessibility of the scapular body. Our measurements have also confirmed this significant variability. The average inclination in relation to the frontal plane is 44.54°, range 21 - 76,9° with a standard deviation of 8.29. In glenoid retroversion, the results range within much smaller limits, with smaller standard deviations. The considerable variability of this inclination may be the result of anatomical conditions (e.g. the shape of the chest, the shape of the scapula, the anatomy and functional status of the muscles surrounding the scapula [21, 22] etc.) or by various pathological states. We chose determination of the scapular inclination angle in relation to the frontal plane for practical reasons. When the patient is lying on the operating table, the frontal plane is visually the simplest reference plane. We are fully aware that scapular inclination may change depending on the patient’s position and on the position of the upper limbs. Nonetheless, the patient’s position on the operating table is very similar to that during the CT examination. The examination took place with the patient supine and their limbs lying freely along the body in a neutral position. When the upper limb is placed in the neutral position during surgery, we obtain minimal errors when determining true glenoid inclination. In the case of using holders with a hole for a scapula may be altered the position and orientation of the scapula. We are also aware of the fact that intraoperatively can change the position of the scapula in the handling of limb. However, we believe that to verify proper components centration will be used drilling of the wire into the center of the glenoid in neutral position of the upper limb. In the neutral position of the limb, the inclination of the scapula can be changed only minimally, while the anatomical variability of the scapula inclination can be significant (Fig. 1).

We believe that awareness of scapular inclination within the framework of preoperative planning may significantly simplify the surgeon’s final assessment of glenoid inclination and thus eliminate potential surgical errors (especially in the case of total shoulder joint replacements [23]). This may have a significant influence of the function and viability of the prosthesis.

Awareness of scapular inclination (especially of the body-neck axis) is also fundamental for the optimal centring of the keel of the glenoid component of a shoulder prosthetic implant. This preoperative knowledge of scapular topography increases in importance especially in conditions involving large defects of the glenoid. In such cases, exact determination of the glenoid version based on its rims is debatable, to say the least.

The main advantage 2D CT is the possibility of precision angular ratios measurement in individual sections [24, 25]. The main advantage of 3D CT is better spatial image of the shape and topography of the individual parts of scapula [24, 25]. Orientation of the glenoid within a 3D space must also be taken into consideration when using various technologies to simulate and navigate scapular orientation within space with the aid of preoperative planning and for the subsequent drawing up of individual surgical templates for prosthetic implants.

Weaknesses of the study is the fact that after the introduction of anesthesia is changed muscle tonus and potentially the orientation of scapula. However, knowledge of the relationship glenoid version and scapula inclination ignores muscle tonus after the release of the glenoid and the determination of the optical retroversion of the glenoid. The limitation of the study is also patient position. Meaning it position on the CT scan in supination is the closest position of the patient on the operating table. Patient lies on back with upper limb loosely at your sides with supination of the forearm.

One of the weaknesses of the study is to conduct a study on the “normal population” (not only in patients with arthritis). But it is necessary to mention that in the file there are many comminuted fractures of the proximal humerus, which were later indicated to hemiarthroplasty or total anatomical arthroplasty of the shoulder. In these patients, it is equally important to know the real orientation of the glenoid for perfect implantation and replacement function as in the revrese shoulder arthroplasty.

## Conclusions

Our measurements have confirmed that glenoid retroversion decreases in the superior-to-inferior direction. We also evaluated the variability of scapular inclination by measuring the thoracoscapular angle. Measurement of the TSa and SIa is a novelty in preoperative planning. However, despite some of those weaknesses will definitely lead to more precise implantation of shoulder arthroplasty (resurfacing, hemiarthroplasty, total anatomical arthroplasty and reverse arthroplasty). We consider the values of the scapular inclination angle (or the thoracoscapular angle) and of glenoid version to be crucial in the preoperative planning of procedures involving the glenoid, especially total shoulder arthroplasty. The reason for this is possible visual interference and incorrect assessment of the true glenoid retroversion during surgery. For example, within the preoperative planning, we found a great scapula inclination (in this case 65 degree (average 45 degree)). This fact helped us extremely in perfect centration of the keel of glenoid component (ventrally drill inclination of approximately 20 degrees) and to the correct determination of the direction of fixing screws for optimal fixation and function of reverse shoulder arthroplasty.

A CT examination is thus necessary during the preoperative workup prior to shoulder arthroplasty. To date, studies have only focused on the variability and significance of glenoid retroversion, however, the true orientation of the glenoid is also affected by scapular inclination (or the thoracoscapular angle). Awareness of the absolute relationship of the anatomical or reverse implant in relation to the body of the scapula and in relation to the chest is equally important. The position of the soft tissues in relation to the prosthesis may then influence the risk of luxation or the predisposition to restriction of rotational movements following arthroplasty. Thus, within the framework of preoperative planning, the surgeon should also take into consideration the value of scapular inclination in order to eliminate potential surgical errors, improve and optimise arthroplasty, and thus decrease the risk of functional restrictions of the joint.

## Abbreviations

SD: Standard deviation
SIa: Scapular inclination angle
TSa: Thoracoscapular angle
VA: Version above
VB: Version below
VM: Version middle
